# Ageing in a variable habitat: environmental stress affects senescence in parasite resistance in St Kilda Soay sheep

**DOI:** 10.1098/rspb.2009.0906

**Published:** 2009-07-08

**Authors:** Adam D. Hayward, Alastair J. Wilson, Jill G. Pilkington, Josephine M. Pemberton, Loeske E. B. Kruuk

**Affiliations:** Institute of Evolutionary Biology, School of Biological Sciences, University of Edinburgh, West Mains Road, Edinburgh EH9 3JT, UK

**Keywords:** ageing, environmental dependence, strongyle helminths, Soay sheep, immunosenescence, faecal egg counts

## Abstract

Despite widespread empirical evidence for a general deterioration in the majority of traits with advancing age, it is unclear whether the progress of senescence is chronologically determined, or whether factors such as environmental conditions experienced over the lifespan are more important. We explored the relative importance of ‘chronological’ and ‘environmental’ measures of age to changes in parasite resistance across the lifespan of free-living Soay sheep. Our results show that individuals experience an increase in parasite burden, as indicated by gastrointestinal helminth faecal egg count (FEC) with chronological age. However, chronological age fails to fully explain changes in FEC because a measure of environmental age, cumulative environmental stress, predicts an additional increase in FEC once chronological age has been accounted for. Additionally, we show that in females age-specific changes are dependent upon the environmental conditions experienced across individuals' life histories: increases in FEC with age were greatest among individuals that had experienced the highest degree of stress. Our results illustrate that chronological age alone may not always correspond to biological age, particularly in variable environments. In these circumstances, measures of age that capture the cumulative stresses experienced by an individual may be useful for understanding the process of senescence.

## 1. Introduction

Biological senescence is a general age-specific decline in physiological condition and fitness (Bonsall 2006). It is manifested in a wide range of traits, from key life-history traits, such as age-specific survival and reproductive performance (Monaghan *et al*. 2008), to aspects of cellular physiology such as telomere length (Monaghan & Haussmann 2006) and oxidative damage (Monaghan *et al*. 2009). The crux of interpreting senescence within an evolutionary framework is that the number of surviving individuals in any cohort decreases with age owing to extrinsic causes of mortality, and so the strength of natural selection declines with age. Theoretically, the onset and rate of senescence can be perfectly predicted by an individual's true ‘biological’ age, an indicator that would predict the ageing state of an individual better than ‘chronological’ age (Klemera & Doubal 2006), which equates to the time since birth in units such as days or years. However, chronological age does not take into account additional environmental factors that may influence the proximate mechanisms of ageing (Monaghan *et al*. 2008), and which may therefore contribute to an individual's biological age. It has been shown in a number of studies that conditions during early growth and development can have profound effects on fitness (e.g. Kruuk *et al*. 1999) and on the trajectory of senescence in later life (e.g. Nussey *et al*. 2007; Reed *et al*. 2008). Environmental conditions can therefore play a large role in determining life-history trajectories, particularly with reference to senescence. In this context, a metric measuring ‘environmental’ age, encompassing the cumulative environmental conditions experienced by an individual across its lifespan, could aid the understanding of senescence-related changes in key life-history traits, especially in free-living systems where individuals are subject to stochastic environments. ‘Biomarkers of ageing’, such as telomere length, hormonal changes and a range of immunological parameters (Simm *et al*. 2008), are thought to provide alternative indicators of biological age, but studying such parameters in the wild has proved to be difficult. It is therefore not clear how well alternative measures of ageing describe changes in performance across an individual's lifetime.

Over the last 20 years, a growing body of work has shown that senescence is pervasive in wild populations (e.g. Jones *et al*. 2008) and occurs in a range of traits in organisms including insects (Bonduriansky & Brassil 2005), fishes (Reznick *et al*. 2004), birds (Gustafsson & Part 1990; Brommer *et al*. 2007; Keller *et al*. 2008) and mammals (Beauplet *et al*. 2006; Nussey *et al*. 2006). Typically, individuals experience declines in survival probability and reproductive performance as they age. Susceptibility to infection also increases, through the process of immunosenescence, an age-specific deterioration in the efficiency of the immune system (Tarazona *et al*. 2002). This subject has received much attention in the laboratory (Gruver *et al*. 2007), but there has been limited work in natural populations and especially in mammals (but see Festa-Bianchet 1989, 1991; Pelletier *et al*. 2005 for a notable example of a longitudinal study of helminth infection in a wild population). Previous work in wild bird populations has proved to be consistent with a decline in aspects of the immune system with age (Cichon *et al*. 2003; Palacios *et al*. 2007). However, such studies are, as far as we are aware, exclusively cross-sectional and as such do not account for the possibility that the observed results are due to individual differences, cohort effects, or interannual variation. In contrast, the use of longitudinal data allows separation of within-individual change from between-individual heterogeneity (Nussey *et al*. 2008).

The Soay sheep population on St Kilda is the subject of one of the world's most intensive longitudinal studies of a free-living mammal population (Clutton-Brock & Pemberton 2004). Data have been collected for over 20 years on population dynamics, individual life histories, parasitism and environmental variables, and provide a unique opportunity to examine the effects of ageing on parasitism. By using individual-based longitudinal data on parasite burdens, we attempted to identify how ageing affects resistance to parasites in this population. We use three separate indicators of biological age: chronological age in years, and two alternative measures indicating an individual's cumulative experience of the environment, which we term environmental age. The first of these sums the number of years of severe mortality an individual has experienced, and the second takes into account environmental conditions experienced in every year of life from birth until sampling to assess the impact of lifetime environmental experience. We also assess how the environmental conditions experienced by individuals across their life histories affect the trajectory of changes in parasitism with chronological age.

Our primary aim was to test, using longitudinal data from a free-living population, for senescence in parasite resistance and to describe age-specific changes in parasitism, with the hypothesis that individuals will experience increasing parasitism as they age. We also test for differences in age-specific parasite infection between the sexes, and predict that males will age more rapidly than females (Clutton-Brock & Isvaran 2007). Secondly, we use chronological and environmental measures of age, and examine how they affect parasitism. Finally, we attempt to identify how age-specific changes are affected by an individual's cumulative environmental experience. We predict that individuals that have experienced poorer environmental conditions will suffer elevated parasitism compared with individuals of the same chronological age that have experienced relatively favourable conditions.

## 2. Methods

### (a) Study population and data collection

**Figure 1.**
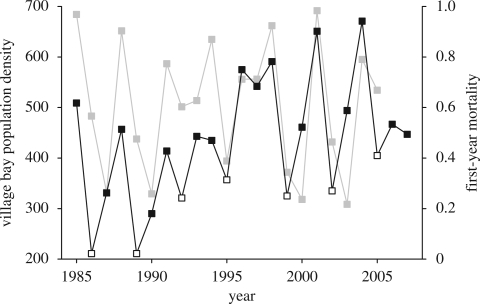
The St Kilda population inhabits a highly variable environment. The system exhibits severe interannual fluctuations in PD (black line), where open symbols show ‘crash years’, and in first-year mortality (grey line), which has varied from 21 to 98 per cent over the course of the study.

The feral Soay sheep population of Hirta (638 ha) in the St Kilda archipelago, NW Scotland (57°49′ N, 08°34′ W) has existed in a free-living state since 1932, when 107 sheep were moved from the neighbouring island of Soay. The current individual-based study began in 1985, since when data have been collected on a range of aspects of the population, including population dynamics, individual life-history and morphological traits and parasitology, as well as a suite of environmental measures (Clutton-Brock & Pemberton 2004). The study focuses on the Village Bay area of the island of Hirta, which contains 200–650 sheep, approximately a third of the island's population. The population exhibits unusual dynamics, with periodic overwinter mortality events (population ‘crashes’) that result in severe reduction in population size (figure 1; Clutton-Brock *et al*. 1991, 1997). Mortality is determined by a combination of population density (PD), demographic structure, winter weather conditions and low food availability (Grenfell *et al*. 1998; Coulson *et al*. 2001).

The sheep are parasitized by a number of parasitic helminth species (Wilson *et al*. 2004), as well as ectoparasites and 13 species of parasitic protozoa (Craig *et al*. 2007). The most prevalent parasite species in the population are the gastrointestinal strongyle nematodes *Teladorsagia circumcincta*, *Trichostrongylus axei* and *Trichostrongylus vitrinus*, infections of which are associated with overwinter mortality (Gulland 1992; Craig *et al*. 2006) and loss of condition as indicated by reduced body weight (Craig *et al*. 2008). Data on infection with these and less-abundant strongyle species, in the form of faecal egg counts (FECs), have been collected since 1988. The McMaster egg counting technique provides an estimate of the number of eggs per gram of faeces, and has been shown to be a good index of parasite burden in Soay sheep, both on St Kilda and elsewhere (Wilson *et al*. 2004). In our analyses, we used strongyle FEC as our response variable for estimating resistance to parasite infection.

### (b) Data and variables

Analysis was performed on data collected between 1985 and 2006, comprising 1806 faecal samples from 227 females and 683 samples from 70 males. This does not represent the total number of FECs available because we removed a proportion of the full dataset for three reasons. First, lambs and yearlings suffer from extremely high parasite burdens before gradually acquiring immunity (Wilson *et al*. 2004; Craig *et al*. 2006), and so we excluded all FECs collected from individuals younger than the age of two. Secondly, during the history of the project, a number of experimental administrations of anthelmintics have been made, and so any samples collected less than a year after anthelmintic treatment were excluded from our analyses. Finally, we only considered individuals that had died, and for which we had complete life-history data.

Chronological age in years was included in all initial models as linear, quadratic and cubic terms, in order to test for a curvilinear effect of age on FEC. We also quantified ‘environmental’ age, which is an individual's experience of the environment and an estimate of the amount of stress it has experienced up to the point of sampling, using two metrics. Our assumption is that conditions experienced immediately before and during a crash are more stressful than those experienced immediately following a crash because adult female sheep show a larger reduction in body weight between autumn and March in crash years (Clutton-Brock *et al*. 1991). The first measure of environmental age was given by the number of winter population crashes an individual has survived (figure 1), with more crashes equating to more stress. Secondly, we used a measure of environmental quality, *E*, which is the proportion of lambs of a cohort surviving for at least 1 year (Wilson *et al*. 2006). This value provides an indicator of environmental quality based on survival of lambs of both sexes. Although the factors influencing lamb survival and adult survival are not identical (Coulson *et al*. 2001), factors that negatively influence lamb survival, such as density and winter weather, have similar effects on female sheep past their reproductive peak and smaller but detectable effects on prime-age adult survival (King *et al*. 2006; Coulson *et al*. 2008). March rainfall is negatively associated with survival in all age and sex classes (Catchpole *et al*. 2000). Our measure of environmental quality, *E*, is negatively associated with PD, winter weather variables and March rainfall (A. Hayward 2009, unpublished data), indicating that environmental conditions affecting adult survival and performance are similar to those affecting senescent and prime-aged female sheep. To gain a measure of environmental stress experienced by an individual, this measure was inverted to give the proportion of lambs dying within a year, and then summed from the time of an individual's birth until the time of sampling, giving a measure of environmental age, cumulative environmental stress (CES). As this measure accumulates with, and positively covaries with, chronological age, we required an alternative measure to predict the influence of environmental conditions across the lifespan on changes in FEC. To remove the colinearity between environmental age and chronological age, we took the mean of the yearly values of inverted *E* and subtracted it from each yearly value. By summing these mean-centred values from birth until the time of sampling, we obtained a measure of the quality of environment experienced over the lifespan that was age-independent, which we refer to as relative environmental stress (RES). Thus, individuals with more positive values of RES have experienced a poorer environment than individuals of the same age with a more negative RES.

An individual's age at death (longevity) was included as a covariate in all of our analyses, in order to account for selective disappearance, the heterogeneity in survivorship of individuals that can produce misleading results in longitudinal analysis of age-specific traits (van de Pol & Verhulst 2006). Intra-annual seasonal environmental conditions are likely to have significant effects on parasite infection, and so we included a number of variables to account for this possibility. Females, in particular, experience a peri-parturient rise, an elevation of parasite burden around the time of offspring birth (Houdijk 2008). We considered season of sampling as a factor with two levels: ‘lambing’ (samples collected in April and May), and ‘other’ (all other months, chiefly August). PD is similarly influential, generally being positively correlated with parasitism (Morand & Poulin 1998), and so we included Village Bay August PD and previous August population density (PPD) as continuous variables. Parasitism is likely to be influenced by climatic conditions, particularly where they have an effect on host condition and survival, as they do in this population (Milner *et al*. 1999). The North Atlantic oscillation (NAO) is a general measure of climatic conditions, with high values indicating warm and wet weather, and low values cool and dry weather, and is commonly used in ecological studies (Stenseth *et al*. 2003). The winter NAO is an average of the monthly NAO values from December to March (Gibraltar–Reykjavik index) and here provides a measure of the climatic conditions during the winter before sampling. Finally, to test for any temporal trend in FEC, and to account for any interannual variation not explained by the specific variables described above, we included year as a continuous covariate in our analyses.

### (c) Statistical analysis

To test for changes in FEC with indicators of age, we used generalized linear mixed-effect models (GLMMs), and all analyses were performed using the GLMM procedure in GenStat 11th edition (VSN International). We used a negative binomial error structure, in order to account for the highly overdispersed nature of parasite data, with few hosts containing the majority of parasites (Wilson & Grenfell 1997). The negative binomial distribution is described by the mean and *k*, a term describing the extent of aggregation (*k* = *μ*^2^/*σ*^2^ – *μ*), and we calculated separate values for both sexes combined (*k* = 0.344), females (*k* = 0.299) and males (*k* = 0.549), indicating that FEC is more uniformly distributed among males. We used a log-link function, estimated the dispersion parameter for each model and used the conditional fitting method of Schall (1991).

In all of our analyses, we included individual identity and year of collection as random effects to account for non-independence of samples taken from the same individual or in the same year. Below, we describe a preliminary model testing for sex-specific differences in patterns of FEC with ageing, and then four subsequent models, each of which were performed on data from both female and male sheep, and which attempt to identify the effects of chronological and environmental measures of ageing.

### (d) Model 0

We pooled data for females and males and constructed a model designed to assess the factors affecting FEC in adults in this population. We investigated sex-specific differences in variables affecting FEC by fitting sex, season, PD, PPD, NAO, year and linear, quadratic and cubic terms for age, as well as interactions between sex and the other variables, where parentheses indicate random effects: FEC*∼*sex *+* longevity *+* season *+* PD *+* PPD *+* NAO *+* year *+* age *+* age^2^ *+* age^3^ *+* sex : longevity *+* sex:season *+* sex : PD*+*sex : PPD *+* sex : NAO *+* sex : year *+* sex : age *+* sex : age^2^ *+* sex : age^3^ +(ID) + (year). This initial model was simplified using the method described below to a final model, which indicated that FEC followed a quadratic trajectory with age (age est. = −0.248 ± 0.069, d.f. = 1, Wald = 4.31, *p* = 0.038; age^2^ est. = 0.024 ± 0.005, d.f. = 1, Wald = 13.49, *p* ≤ 0.001). There was also a significant interaction between sex and age (male est. = 0.106 ± 0.043, d.f. = 1, Wald = 6.15, *p* = 0.013). Inspection of the parameter estimates reveals that FEC remains effectively constant in males aged 2 to 4, and then increases, with the highest FEC in the oldest sheep at age 8. In females, FEC is predicted to initially decline with age, reaching a trough around the age of 5 or 6, before subsequently increasing from the age of 10 onwards. This result, coupled with the differences between the sexes in biology, longevity, parasite aggregation and age distribution of the data, encouraged us to separate the sexes for subsequent analyses.

### (e) Model 1

Having established that age-specific changes in FEC differ between the sexes, we described the relationship between chronological age and FEC in each sex separately with a model incorporating current environmental factors and chronological age: FEC ∼ longevity + season + PD + PPD + NAO + year + age + age^2^ + age^3^ + (ID) + (year).

These models allowed us to fit sex-specific aggregation parameters to represent the differences in the distribution of FEC between the sexes.

### (f) Model 2

Our second model assessed changes in FEC with the number of population crashes experienced by an individual, as a crude measure of environmental age, while controlling for chronological age. Note that AGE indicates the linear and quadratic chronological age terms in females, and solely the linear term in males, because these were the variables that emerged from model 1 in females and males, respectively: FEC ∼ longevity + season + PD + PPD + NAO + year +AGE + crashes + (ID) + (year).

### (g) Model 3

Thirdly, we employed our cumulative measure of environmental age, CES, as an alternative to the number of population crashes experienced, in order to further assess changes in FEC with environmental age, while controlling for chronological age: FEC ∼ longevity + season + PD + PPD + NAO + year + AGE + CES + (ID) + (year).

### (h) Model 4

Finally, to assess the influence of environmental experience onchanges in FEC with chronological age, we used a model with an interaction between RES and chronological age: FEC ∼ longevity + season + PD + PPD + NAO + year + AGE + RES + AGE : RES + (ID) + (year).

All initial models were simplified until only significant variables, or those involved in significant interactions, remained. Significance of fixed effects was assessed using Wald statistics and associated conditional *p*-values with the appropriate degrees of freedom.

## 3. Results

### (a) Model 1

**Figure 2.**
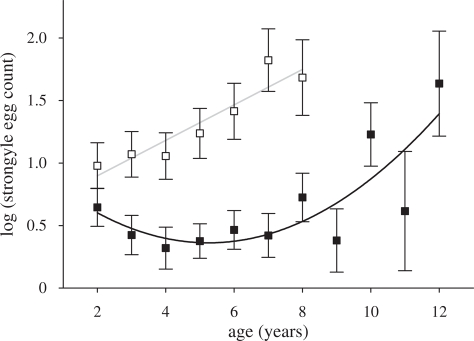
Model predictions of age-specific means and SEs of FEC. Female sheep (filled symbols, black line) experience enhanced resistance to parasitism until the age of five, and experience a subsequent increase thereafter. On the other hand, males (open symbols, grey line) show a linear increase in strongyle FEC from the age of two onwards, and maintain higher FEC than females across life.

In female sheep, there was a significant quadratic effect of age on FEC (est. = 0.025 ± 0.001SE, Wald = 19.55, d.f. = 1, *p* ≤ 0.001; figure 2), indicating a decline in FEC from the age of 2 until the age of 5, followed by a rapid increase in later life. Male sheep, on the other hand, showed a significant linear increase in FEC from the age of 2 onwards (age est. = 0.127 ± 0.036, Wald = 12.54, d.f. = 1, *p* ≤ 0.001; figure 2). Hence, there is evidence to suggest that Soay sheep underwent a senescent decline in the ability to resist parasite infection, but that this decline contrasts between sexes, as predicted by the preliminary results from model 0.

### (b) Model 2

**Figure 3.**
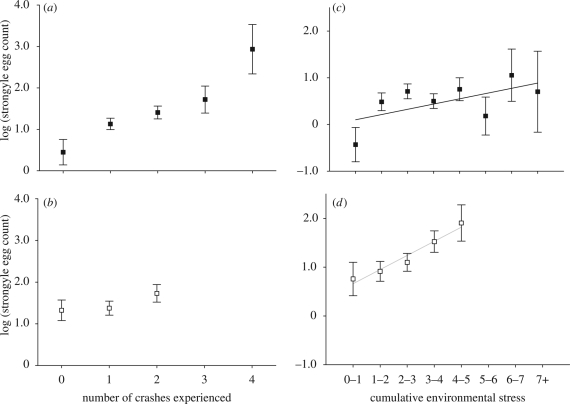
Model predicted means and SEs for age-specific changes in FEC for two measures of environmental age. (*a*) Females show a significant increase in FEC with the number of population crashes experienced, and this effect is most marked in the oldest individuals; (*b*) males show a non-significant increase in FEC as they experience more population crashes; (*c*) females show a highly significant linear increase in FEC with accumulating environmental stress; (*d*) males show a marginally non-significant linear increase with increasing experience of environmental stress.

The final model in females incorporating the number of crashes experienced showed a significant effect of number of crashes experienced on FEC (Wald = 15.02, d.f. = 4, *p* = 0.005; figure 3*a*). Thus, in a model controlling for chronological age, females experienced an increase in FEC with increasing environmental age. The effect of crashes remains highly significant if chronological age is omitted from the model (Wald = 32.17, d.f. = 4, *p* ≤ 0.001). The final model for males did not show a significant increase in FEC with increasing environmental age (Wald = 4.65, d.f. = 2, *p* = 0.099; figure 3*b*).

### (c) Model 3

We next fitted a model with CES as a measure of environmental age, again controlling for chronological age. We found a significant positive linear relationship between CES and FEC in females, indicating that individuals that have experienced a higher degree of environmental stress suffered from increased parasitism (table 1; figure 3*c*). This effect remains if chronological age is dropped from the model (est. = 1.280 ± 0.047, Wald = 7.45, d.f. = 1, *p* = 0.007). However, males exhibited a marginally non-significant positive effect of CES on FEC (table 2; figure 3*d*). As with the number of crashes experienced, this is suggestive of a similar process to that occurring in females, yet is not supported by statistical significance.

**Table 1.** Results of the final minimal GLMM showing the effect of environmental age on strongyle FEC in female sheep.

variables	estimate	SE	d.f.	Wald	*p*-value
*fixed effects*					
intercept	1.193	0.128			
longevity	−0.113	0.027	1	15.03	<0.001
season					
lambing	0.000	0.079	1	297.86	<0.001
other	−1.438				
PPD	0.002	0.001	1	5.67	0.028
NAO	0.170	0.064	1	8.77	0.005
age	−0.632	0.114	1	2.56	0.110
age^2^	0.021	0.006	1	20.87	<0.001
CES	0.723	0.173	1	17.50	<0.001
*random effects*					
ID	0.500	0.079			
year	0.136	0.068			

**Table 2.** Results of the final minimal GLMM model showing the effect of environmental age on strongyle FEC in male sheep.

variables	estimate	SE	d.f.	Wald	*p*-value
*fixed effects*					
** **intercept	1.513	0.195			
** **longevity	−0.128	0.039	1	4.21	0.045
** **season					
lambing	0.000	0.144	1	18.50	<0.001
other	−0.657				
** **NAO	0.226	0.082	1	5.54	0.026
** **age	−0.056	0.105	1	12.59	<0.001
** **CES	0.315	0.170	1	3.43	0.065
*random effects*					
** **ID	0.255	0.073			
** **year	0.153	0.079			

A further note on these models is that parameter estimates for chronological age from models with and without CES are inconsistent across models. In female model 1, chronological age has a quadratic effect on FEC, describing a decrease and a subsequent increase in FEC with age. However, in female model 3, the quadratic effect of age describes a decelerating decline with increasing chronological age (age est. = −0.632 ± 0.114; age^2^ est. = 0.021 ± 0.006, Wald = 20.87, d.f. = 1, *p* ≤ 0.001). While there is colinearity between these two variables, the effect of CES does not change whether or not age is accounted for, and so here is a robust indicator of changes in FEC.

### (d) Model 4

**Figure 4.**
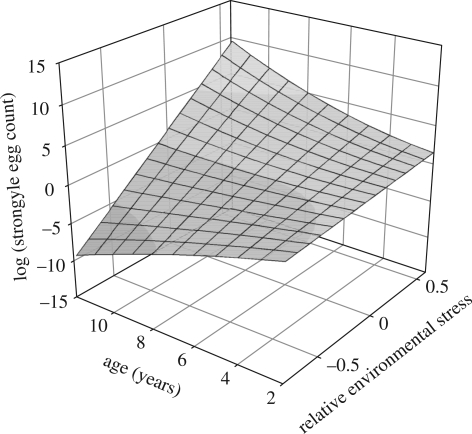
Female sheep experiencing a higher degree of environmental stress across their life spans exhibit higher FEC at a given age than female sheep experiencing lower stress. At low levels of environmental stress, FEC decreases with chronological age, but this pattern is reversed in females enduring higher levels of stress.

Finally, an interaction model attempting to identify an effect of environmental experience on age-specific changes in FEC yielded a significant interaction between age^2^ and RES in females (est. = 0.052 ± 0.021, Wald = 6.36, d.f. = 1, *p* = 0.012; figure 4), indicating that the change in FEC with age changes from negative to positive with increasing stress suffered. Therefore, at low stress, FEC decreases with age, while at high stress it increases with age. The trajectory of age-specific changes in FEC with age is, therefore, dependent on an individual's experience of the environment over its life history. In males, the interaction between chronological age and RES was non-significant (est. = −0.125 ± 0.103, Wald = 1.49, d.f. = 1, *p* = 0.223), suggesting that FEC in males is independent of environmental conditions experienced over the lifespan.

### (e) Other variables

Tables 1 and 2 show other variables that were found to influence FEC in all models in females and males, respectively. Both sexes experienced significantly higher FEC during the lambing season and following winters with high NAO values, indicating warmer and wetter weather. Longevity was negatively associated with FEC in both females and males, indicating that longer lived sheep generally exhibit lower FEC and justifying our attempts to control for selective disappearance. Finally, previous summer's PD was positively associated with FEC in females, but not males, indicating that transmission events occurring prior to winter may influence worm burden during the following year. This demonstrates that specific environmental effects influence parasitism and that simply accounting for interannual variation by including year as a covariate or random effect may not be sufficient in this respect.

## 4. Discussion

We have presented results showing that, as predicted by previous work on immunosenescence, feral Soay sheep experience declining ability to resist parasite infection as they age chronologically. We have also shown that an alternative measure of ageing, environmental age, also predicts an increase and that chronological age alone is insufficient to describe senescence in this context. Finally, we have demonstrated that the nature of changes in parasitism with age is highly dependent upon the environmental conditions individuals experience across their life histories.

Many life-history traits measured in wild populations can be well explained as a quadratic function of chronological age, with an improvement in the trait from early life until a peak in ‘prime age’, followed by a senescent decline in later life (Jones *et al*. 2008). The bulk of studies of immunosenescence either compare age classes (e.g. Saino *et al*. 2003) or show a linear decline in immunological parameters with age (e.g. Haussmann *et al*. 2005; Palacios *et al*. 2007), which therefore contrast with the quadratic form described in studies of other traits. A decrease in assayable immune parameters does not necessarily predict an increase in parasitism with senescence because an optimal immune response is not necessarily the strongest possible (Viney *et al*. 2005), but in both female and male sheep, we show that there is an increase in parasitism with chronological age, suggesting senescence in the efficiency of the immune response to helminth infection. Moreover, it appears that the age-related increase in FEC begins from age 8 in females and age 5 in males (figure 2). This represents data on 93 different females (41% of the individual females in our dataset) and 31 different males (44% of individual males in our dataset), indicating that a substantial proportion of individuals surviving to adulthood reach an age at which they experience increasing parasite burden as they get older. This is, as far as we are aware, the first longitudinal analysis of senescence-related changes in parasite infection or resistance.

Our two indicators of environmental age, number of crashes experienced and CES, both predicted an increase in FEC, and these results also hold whether or not chronological age was included in the model. This indicates that chronological age alone does not describe senescence-related changes in FEC and that incorporating a measure of environmental age in addition to chronological age can provide more information about the process of senescence.

By using RES as a relative measure of environmental age, we have shown that conditions experienced throughout life can have a profound impact upon age-specific changes in an important fitness trait, namely FEC, an estimate of parasite burden and resistance. Thus, when experiencing low levels of environmental stress, females showed an improved ability to resist parasites with age, as estimated by falling FEC. However, when experiencing relatively poor conditions over their lives, females showed a progressively faster increase in FEC with age. This shows that environmental conditions can have a profound impact upon rates of senescence. The results of analyses on males suggested that changes in FEC with chronological age seemed to be independent of RES. A possible explanation could be simply that we lacked the statistical power to detect any interaction, though this is unlikely given that standard errors were lower for males than for females. A second explanation is that the more rapid life history of males (Clutton-Brock *et al*. 2004) makes them less vulnerable to the cumulative effects of environmental stress because the majority may not live long enough to express its effects. Similarly, because of the lower life expectancy of males, they do not experience the same range of CES as females and so either do not express its effects or do so only weakly.

Although we cannot identify the proximate mechanisms driving these changes, we can comment in broad terms on how cumulative exposure to environmental stress may affect parasite resistance. One possibility is that experience of adverse environmental conditions has irreparable effects on physiology, which are proportional to the cumulative amount of stress suffered. For instance, limited resources in poor conditions may be shifted away from immunocompetence and into maintenance of body weight or to a developing foetus, which may explain the effects of PPD and NAO on the FEC we have shown here. Persistent experience of poor environments and parasite infection may have an adverse effect on the ability of the immune system to respond to infections in later life, as the proliferative capability of T cells becomes exhausted (Akbar *et al*. 2004; Vleck *et al*. 2007). It has also been shown that strongyle infection can cause physical damage to the sheep abomasum (Gulland 1992), and such damage accumulated over time could have adverse effects on the ability to assimilate nutrients and, therefore, maintain an effective immune system. A final possibility is that sheep experiencing poorer cumulative environments have faced greater exposure to parasites than other sheep of the same age, and so, for instance, sheep with a higher RES for a given age may simply express past exposure, rather than current state, in higher FEC. As current state depends on the past experience of environmental conditions, it would be extremely difficult to separate these effects.

This longitudinal study suggests that senescence-related changes in parasite resistance are dependent upon the environmental conditions experienced over the lifetime of an individual. We are unable here to identify the mechanistic nature of the relationship between parasitism, ageing and the immune system, and so a fruitful avenue of future research will be to characterize immunosenescence in a longitudinally monitored natural population and to relate this to actual parasite burdens. We have used a ubiquitous indicator of environmental quality, namely the proportion of first-year mortality, and so identifying specific environmental variables that influence senescence-related changes provides a challenge for further research in this and in other systems. A direct and intuitive route from the current study would be to expand the analyses herein to investigate occurrences at different stages of the life history of individuals and their effects on ageing and parasitism. Conditions experienced during early development, either pre- or postnatally, could contribute to age-specific changes in the same way as environmental ageing. Further, parasitism during development could affect later-life changes in other life-history traits. In relatively constant environments, chronological and environmental age will be virtually equivalent because damage or stresses caused by environmental conditions will accumulate at a constant rate. Our current results indicate that in assessing any such impacts on life histories, the environmental conditions an individual experiences across its lifespan may play a significant role in determining rates of ageing, and that these impacts will be particularly noticeable in variable environments.
